# Elucidation of molecular interactions of theaflavin monogallate with camel milk lactoferrin: detailed spectroscopic and dynamic simulation studies†

**DOI:** 10.1039/d1ra03256a

**Published:** 2021-08-04

**Authors:** Mohd Shahnawaz Khan, Rais Ahmad Khan, Md Tabish Rehman, Mohamed A. Ismael, Fohad Mabood Husain, Mohamed F. AlAjmi, Majed S. Alokail, Nojood Altwaijry, Ali M. Alsalme

**Affiliations:** Protein Research Chair, Department of Biochemistry, College of Science, King Saud University Riyadh 11451 Saudi Arabia moskhan@ksu.edu.sa; Department of Chemistry, College of Science, King Saud University Riyadh Saudi Arabia; Department of Pharmacognosy, College of Pharmacy, King Saud University Riyadh Saudi Arabia; Department of Food Science and Nutrition, College of Food and Agriculture Science, King Saud University Riyadh Saudi Arabia

## Abstract

Lactoferrin is a heme-binding multifunctional glycoprotein known for iron transportation in the blood and also contributes to innate immunity. In this study, the interaction of theaflavin monogallate, a polyphenolic component of black tea, with camel milk lactoferrin was studied using various biophysical and computational techniques. Fluorescence quenching at different temperatures suggests that theaflavin monogallate interacted with lactoferrin by forming a non-fluorescent complex, *i.e.*, static quenching. Theaflavin monogallate shows a significant affinity towards lactoferrin with a binding constant of ∼10^4^–10^5^ M^−1^ at different temperatures. ANS binding shows that the binding of polyphenol resulted in the burial of hydrophobic domains of lactoferrin. Moreover, thermodynamic parameters (Δ*H*, Δ*S* and Δ*G*) suggested that the interaction between protein and polyphenol was entropically favored and spontaneous. Circular dichroism confirmed there was no alteration in the secondary structure of lactoferrin. The energy transfer efficiency (FRET) from lactoferrin to theaflavin was found to be approximately 50%, with a distance between protein and polyphenol of 2.44 nm. Molecular docking shows that the binding energy of lactoferrin–theaflavin monogallate interaction was −9.7 kcal mol^−1^. Theaflavin monogallate was bound at the central cavity of lactoferrin and formed hydrogen bonds with Gln89, Tyr192, Lys301, Ser303, Gln87, and Val250 of lactoferrin. Other residues, such as Tyr82, Tyr92, and Tyr192, were involved in hydrophobic interactions. The calculation of various molecular dynamics simulations parameters indicated the formation of a stable complex between protein and polyphenol. This study delineates the binding mechanism of polyphenol with milk protein and could be helpful in milk formulations and play a key role in the food industry.

## Abstract 2

Lactoferrin is a heme-binding multifunctional glycoprotein known for iron transportation in the blood and also contributes to innate immunity.

## Graphical abstract

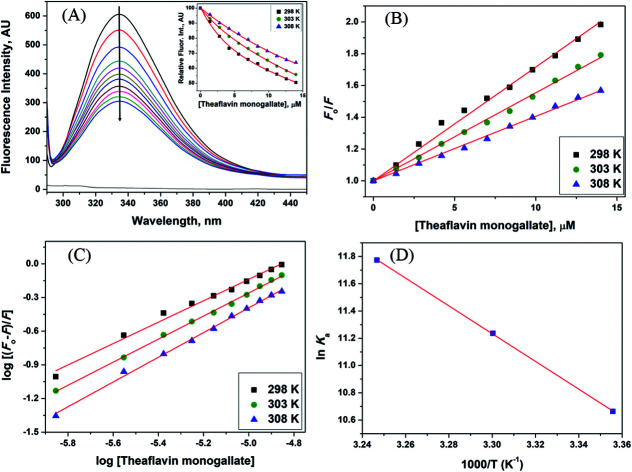

## 1. Introduction

Lactoferrin (MW = ∼80 kDa) is an iron-binding protein present in mammalian milk and other exocrine secretions such as tears, nasal and bronchial mucous and saliva.^1,2^ It belongs to the transferrin family of non-heme protein. Lactoferrin plays various roles in the innate immunology of the host,^3^ inhibition of neutrophil priming by bacterial lipopolysaccharide,^4^ and modulating inflammation by amplifying apoptotic signals.^5^ Moreover, lactoferrin has also been documented to exhibit anti-tumor, anti-fungal, anti-viral, and anti-bacterial properties.^6^ Lactoferrin has recently come under the spotlight, particularly with regards to the new coronavirus pandemic that started in 2019 (COVID-19). A recently published literature study suggested that lactoferrin can bind to at least some of the receptors used by coronaviruses thereby blocking their entry.^7^

Structurally, a simple polypeptide chain of lactoferrin is folded to form two symmetrical lobes. These two lobes (N and C) are highly homologous of approximately 33–41%.^8^ The N lobe of this polypeptide chain ranges from 1–332 amino acids, and C lobes range from 344–703 amino acids. Both these lobes are comprised of α-helices and β-pleated sheets, creating two domains for each lobe *viz.* domains I and II.^9^ These lobes are connected by a hinge region which provides additional flexibility to the protein.^10^ Both lobes adopt open conformations indicating wide distances between the iron binding residues in the native iron-free form of lactoferrin. The structure of human apolactoferrin was more intriguing in which the N-lobe adopted an open conformation while the C-lobe stayed in the closed conformation similar to its holo-form. Bovine and human lactoferrins can bind to the HCV envelope proteins E1 and E2. This binding inhibits any possible interaction of the virus with its cellular receptors. Similar results have recently been reported for camel lactoferrin, demonstrating complete inhibition of virus entry when camel lactoferrin and HCV were preincubated together. In contrast, lactoferrin pre-incubation with human leukocytes, HepG2 cells and Huh7.5 cells before HCV infection did not affect viral entry.^11^ Moreover, in comparison to cow milk, camel milk is rich in vitamin C, niacin, vitamin A and E.^12,13^ Camel milk has a high content of α-lactoalbumin and lactoferrin but lacks β-lactoglobulin.^14^ In the past, camel milk has been used as a nutritional supplement in chronic pulmonary tuberculosis.^15^ It has no allergenic properties and can be taken by lactase-deficient and immune-deficient people.^16^

Theaflavins are a class of polyphenols and is a major component of black tea. They are regarded as ‘golden molecules’ owing to their therapeutic attributions.^17^ They have been shown to have various physiological actions, including antioxidant,^18^ anticancer,^19^ anti-atherosclerotic,^20^ anti-inflammatory,^21^ antiviral,^22,23^ anti-periodontitis^24^ and the inhibition of osteoporosis.^25^ Furthermore, these compounds have been shown to possess human health benefits including glucose-lowering^26^ anti-obesity^27^ as a prevention of lifestyle-related diseases.

Studies delineating the interaction of clinically significant proteins with natural compounds are on a high in present times owing to the fact natural compounds possesses enormous structural and chemical diversity^28^ coupled with their clinical potential thereby playing a key role in drug discovery.^29^ Previous literature suggested the superiority of camel milk and the use of lactoferrin in various marketing products such as infant formulas, probiotics, supplemental tablets, cosmetics and as a natural solubilizers of iron in food^30^ prompted us to explore its interactions with theaflavin monogallate. Several biophysical and computational tools were performed to reveal the conformational changes and mechanism of binding between protein–polyphenols complex.

## 2. Material and methods

### 2.1. Materials

Lactoferrin from camel milk was isolated and purified from a previous procedure.^31^ Theaflavin monogallate and ANS (8-anilinonaphthalene-1-sulfonic acid) dye were obtained from Sigma-Aldrich (MO, USA). All other chemicals were of a high standard and analytical grade. A stock solution of ANS was prepared in double-distilled water. The stock solution of theaflavin monogallate was prepared in DMSO. The concentration of DMSO used in the experiment was less than 1%.

### 2.2. Steady-state fluorescence study

The steady-state fluorescence was performed using a spectrofluorometer (Jasco FP-750, Japan). Briefly, lactoferrin (5 μM) was excited at 280 nm, and its fluorescence emission spectrum was recorded from 290 to 450 nm. Theaflavin monogallate (0–15 μM) was titrated to lactoferrin solution, and fluorescence emission spectra were recorded at each titration. The experiment was performed at 298, 303, and 308 K for the analysis of thermodynamic parameters. The fluorescence of the buffer solution was subtracted from the fluorescence spectra of lactoferrin. Moreover, the inner filter effect on the fluorescence was corrected using the following relation (eqn (1)).

1 *F*_c_ = *F*_o_*e*^(*A*_ex_+*A*_em_)/2^

where *F*_c_ and *F*_o_ are the corrected and measured fluorescence, respectively; *A*_ex_ and *A*_em_ are the absorptions of protein and ligand complex at the excitation and emission wavelengths, respectively.

### 2.3. Circular dichroism measurements

The circular dichroism (CD) measurements were carried out using Applied Photophysics, ChirascanPlus, UK spectropolarimeter attached with Peltier. The CD spectra of native lactoferrin (5 μM) and their complex with theaflavin monogallate (1 : 5 and 1 : 10 protein : ligand ratio) were recorded in the far-UV range between 200–250 nm. Sodium phosphate buffer (20 mM, pH 7.4) was used for the baseline correction. Each spectrum presented is the average of 3 scans.

### 2.4. Hydrophobicity analysis using ANS

The hydrophobicity of lactoferrin in the presence of varying concentrations of theaflavin monogallate was determined by measuring ANS fluorescence. To the fixed concentration of lactoferrin (5 μM), a 50 times higher concentration of ANS dye was added. The fluorescence emission spectrum was recorded for native lactoferrin by exiting at 380 nm. Further, theaflavin monogallate (0–15 μM) was titrated to the solution (protein–ANS), and the emission signals were obtained from 400 to 650 nm. All measurements were carried out at 298 K.

### 2.5. Forster resonance energy transfer (FRET)

The UV-visible absorption spectrum of theaflavin monogallate (10 μM) was recorded using UV-vis Spectrophotometer (Amersham Bioscience, Sweden). The fluorescence emission of spectra of lactoferrin (5 μM) was recorded by exciting at 295 nm. All the fluorescence and absorbance readings were normalized to 1. The overlap between the fluorescence spectrum of lactoferrin and the absorption spectrum of the theaflavin monogallate was determined, and the FRET parameters were determined as reported previously.^32^

### 2.6. Molecular docking analysis

The interaction between lactoferrin and theaflavin monogallate was elucidated by performing molecular docking using AutoDock4.2, as described previously.^31^ The three-dimensional coordinates of camel lactoferrin (PDB Id: 1I6Q, resolution: 2.70 Å) were obtained from the RSCB database, while the structure of theaflavin monogallate was obtained from PubChem (CID: 169167). Before molecular docking, lactoferrin's structure was optimized by deleting crystallographic water molecules and any other heteroatoms, adding missing hydrogen atoms, and defining Kollman united atom type charge using AutoDock Tool (ADT). Theaflavin monogallate was pre-processed by defining rotatable bonds, number of torsions, and adding Gasteiger partial charges. The energy of the theaflavin monogallate was minimized using a universal force field (UFF). A grid map of 103 × 76 × 71 Å centered at 39.8 × 1.9 × 8.4 Å with 0.375 Å spacing was created using AutoGrid. The parameters in AutoDock were set to their default values, and distance-dependent dielectric parameters were employed to calculate van der Waals' and electrostatic energies. Lamarck Genetic Algorithm (LGA) along with Solis and Wets methods, were employed to perform docking calculations. For each run, a maximum of 2 500 000 energy terms was calculated, and a total of 10 runs were computed. The initial position, torsion, and orientation of theaflavin monogallate were set randomly. The population size, translational step, quaternions, and torsions were set to 150, 0.2 Å, and 5, respectively. The binding affinity (*K*_d_) of theaflavin monogallate towards lactoferrin was calculated from binding energy (Δ*G*) using the following relation.^32^

2 Δ*G* = −*RT* ln *K*_d_

where *R* and *T* were the Boltzmann gas constant and temperature, respectively.

### 2.7. Molecular dynamics (MD) simulations

The stability and dynamics of the lactoferrin–theaflavin monogallate complex were evaluated by performing MD simulation using GROMACS version 2020.2, as described earlier.^33^ The topology of lactoferrin was generated using the pdb2gmx command and selecting OPLS (Optimized Parameters for Liquid Simulation) forcefield. Conversely, the topology parameters of theaflavin monogallate were generated in the LigParGen server and combined with the lactoferrin topology. The lactoferrin and lactoferrin–theaflavin monogallate complex systems were placed in a cubic box and solvated with an spc216 water model to mimic the aqueous environment. The energies of both the systems were minimized for 1000 ps using 1500 steps of the steepest descent method. The temperature of both the systems was gradually increased under periodic boundary conditions from 0 to 300 K through an equilibration period of 100 ps at constant volume and pressure (1 bar). For both the systems, the final production MD run was performed for 50 ns, and the resulting trajectories were analyzed as described previously.^33^

## 3. Results and discussion

### 3.1. Steady-state fluorescence

**Fig. 1:**
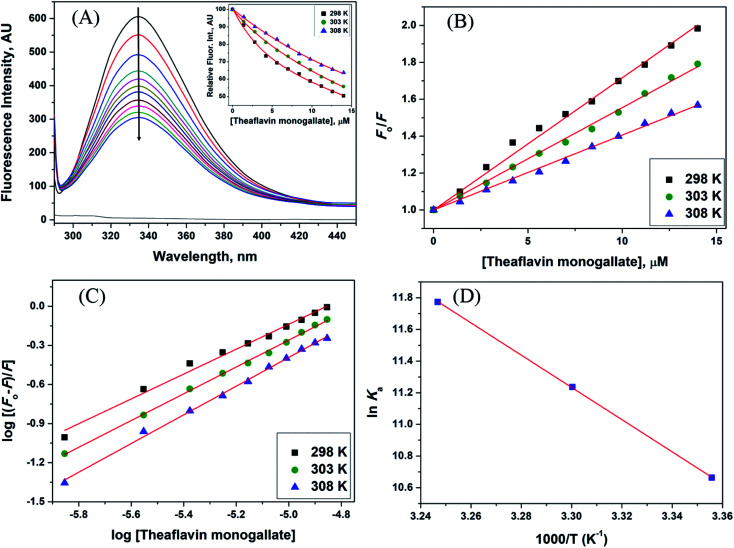
Interaction between lactoferrin and theaflavin monogallate. (A) Quenching in fluorescence intensity of lactoferrin (5 μM) in the absence and presence of varying theaflavin monogallate concentration (0–15 μM) at 298 K, (B) Stern–Volmer plot at different temperatures, (C) modified Stern–Volmer plot at different temperatures, and (D) van't Hoff thermodynamics plot at three different temperatures.

The steady-state fluorescence spectroscopy is a valuable tool to study proteins' interaction with small molecules or drugs. In this technique, small molecules' effect on the native fluorescence emission spectrum of protein is analyzed. The fluorescence emission signal of camel milk lactoferrin in the absence and presence of varying concentrations (0.5–15 μM) of theaflavin monogallate is shown in Fig. 1A. The fluorescence quenching of lactoferrin in the presence of theaflavin monogallate at temperatures 303 and 308 K is also shown in ESI, Fig. 1.† Native lactoferrin exhibited fluorescence maxima at 335 nm, a characteristic signal of lactoferrin protein.^34^ The addition of theaflavin monogallate resulted in a progressive quenching of lactoferrin fluorescence signal, indicating an interaction between protein and polyphenol.^35^ Moreover, theaflavin monogallate just caused the quenching without changing the emission maximum and shape of the peak. The similar trend was earlier observed in BSA protein after interaction with theaflavins.^36^ Further, quenching fluorescence of lactoferrin–theaflavin monogallate binding was mathematically evaluated using Stern–Volmer eqn (3) to obtain *K*_sv_.^37^3
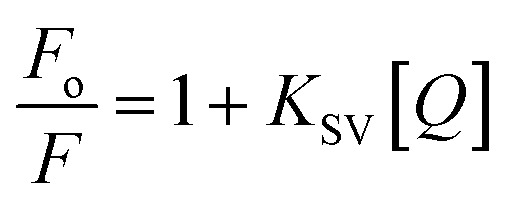
where *F*_o_ is the fluorescence maxima of free or native lactoferrin; *F* is the fluorescence maxima of lactoferrin in the presence of theaflavin monogallate; *K*_SV_ is Stern–Volmer constant, and [*Q*] is the concentration of theaflavin monogallate.

The Stern–Volmer plot for the interaction of theaflavin monogallate with lactoferrin is shown in Fig. 1B, and the values of *K*_SV_ are illustrated in Table 1. The *K*_SV_ values for the interaction of theaflavin monogallate with lactoferrin were 7.16 × 10^4^, 5.54 × 10^4^, and 4.06 × 10^4^ M^−1^ at 298, 303, and 308 K, respectively. However, the values of *K*_SV_ alone cannot ensure the mode of fluorescence quenching. To analysis the mode of fluorescence quenching, bimolecular quenching rate constant (*k*_q_) using eqn (4).^38^4
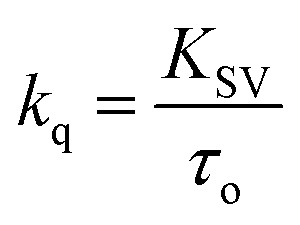
where *τ*_0_ is the fluorescence lifetime whose value is approximately 5.78 × 10^−9^ s. The values of *k*_q_ as a function of temperature varied in the range of 0.71–1.25 × 10^13^ M^−1^ s^−1^ (Table 1). The bimolecular quenching rate constant (*k*_q_) for all tested system are higher than the maximum scatter collision constant (2 × 10^10^ M^−1^ s^−1^ for dynamic quenching).^38^ Thus, our results illustrated the static mode of quenching in lactoferrin fluorescence occurred due to the formation of a non-fluorescent complex between theaflavin monogallate and lactoferrin. The quencher physically interacts with the excited molecule through chemical bonds in static quenching.^40^ The mode of quenching can further be confirmed by analyzing the dependence of *K*_sv_ on temperature. The values of *K*_q_ and *K*_sv_ (Table 1) were inversely correlated with temperature for theaflavin monogallate, which again indicated that the nature of quenching was static. Earlier reports also validated the static mode of quenching between polyphenols and proteins.^36,39^ In static quenching, *K*_sv_ decreases with increasing temperature due to the breakdown of weakly bound complexes. However, in dynamic quenching, *K*_sv_ increases with an increase in temperature due to a higher diffusion rate.^41^

**Table 1:** Fluorescence quenching parameters for camel milk lactoferrin and theaflavin monogallate interaction

Temp. (K)	*K*_SV_ × 10^4^ (M^−1^)	*k*_q_ × 10^13^ (M^−1^ s^−1^)	*K*_a_ × 10^4^ (M^−1^)	*n*
298	7.16	1.25	4.28	0.9538
303	5.54	0.97	7.58	1.0279
308	4.06	0.71	12.97	1.1010

### 3.2. Determination of binding parameters

The fluorescence quenching data was further used to determine the binding constant (*K*) and number of binding sites. These binding parameters were calculated using the modified Stern–Volmer eqn (5).^42^5
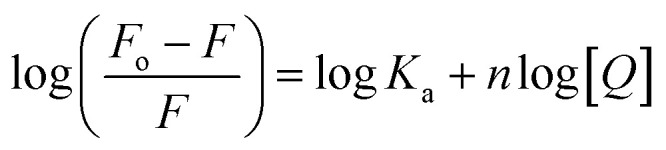
where *K*_a_ is the association, or binding constant, and *n* is the number of binding sites. The modified Stern–Volmer plot is shown in Fig. 1C, and the values obtained is enlisted in Table 1. The values *K*_a_ were found to be 4.28 × 10^4^, 7.58 × 10^4^, and 12.97 × 10^4^ M^−1^ at 298, 303, and 308 K, respectively. These values in the order of 10^4^ M^−1^ suggested that theaflavin monogallate binds with significant affinity to lactoferrin. The number of binding sites was estimated to be close to 1, signifying that there was only one binding site of theaflavin monogallate on lactoferrin. Previously, EGCG and theaflavins were reported to possess single binding domain with albumin.^36^ Moreover, there was an increase in binding constant values with increasing temperature, showing that the protein–polyphenol complex was relatively more stable at higher temperatures.

### 3.3. Binding thermodynamics: lactoferrin–theaflavin interactions

The values of binding constant at different temperatures were used for the calculation of thermodynamic parameters. The nature of forces or type of bonds responsible for the complexation of theaflavin monogallate to lactoferrin was determined using thermodynamic parameters. The active force between phenolic compounds and biomolecules may include electrostatic interactions, van der Waals interactions, and hydrophobic effect and so on. The model of interaction between quencher and a protein molecule can be concluded according to Δ*H*^0^ and Δ*S*^0^ data.^43^ More specifically, (1) Δ*H*^0^ > 0 and Δ*S*^0^ > 0, the main force would be hydrophobic (2) Δ*H*^0^ < 0 and Δ*S*^0^ > 0, it would be electrostatic force; (3) Δ*H*^0^ < 0 and Δ*S*^0^ < 0, it would be hydrogen bond and van der Waals interactions.^36,44,45^ The values and the mathematical sign of thermodynamic parameters provide insight regarding the various interactions. The values of entropy change (Δ*S*) and enthalpy change (Δ*H*) were obtained using van't Hoff's eqn (6).^46,47^6
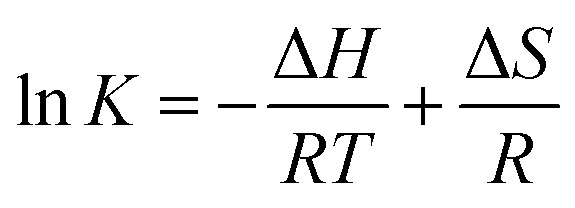
where *T* is temperature and *R* is the universal gas constant (1.987 cal mol^−1^ K^−1^). The van't Hoff's plot is shown in Fig. 1D, and values of Δ*S* and Δ*H* are presented in Table 2. The positive value of entropy change suggests that water molecules were arranged in an orderly fashion around the lactoferrin that got randomized after the interaction of theaflavin monogallate.^45^ A positive value of enthalpy change confirms that the interaction of theaflavin monogallate with lactoferrin was endothermic. Moreover, the positive values of both entropy and enthalpy change indicate the formation of hydrophobic interaction between lactoferrin and theaflavin monogallate.^48^ The value of Gibb's free energy change (Δ*G*) was calculated using eqn (7).

7 Δ*G* = Δ*H* − *T*Δ*S*

**Table 2:** Thermodynamics parameters for the interaction between camel milk lactoferrin and theaflavin monogallate

Temp. (K)	Δ*H* (kcal mol^−1^)	Δ*S* (cal mol^−1^ K^−1^)	*T*Δ*S* (kcal mol^−1^)	Δ*G* (kcal mol^−1^)
298	20.25	89.13	26.56	−6.31
303			27.01	−6.76
308			27.45	−7.20

The values of Δ*G* at all tested temperatures were negative and ranged from −7.20 to −6.31 kcal mol^−1^ (Table 2). The negative value of Δ*G* confirms the binding of lactoferrin with theaflavin monogallate to be a spontaneous reaction.^46^ Based on the sign and values of these binding parameters, various literature suggested the hydrophobic forces played a significant role in the bindings of polyphenols–protein.^36,49^

### 3.4. Circular dichroism spectroscopy

**Fig. 2:**
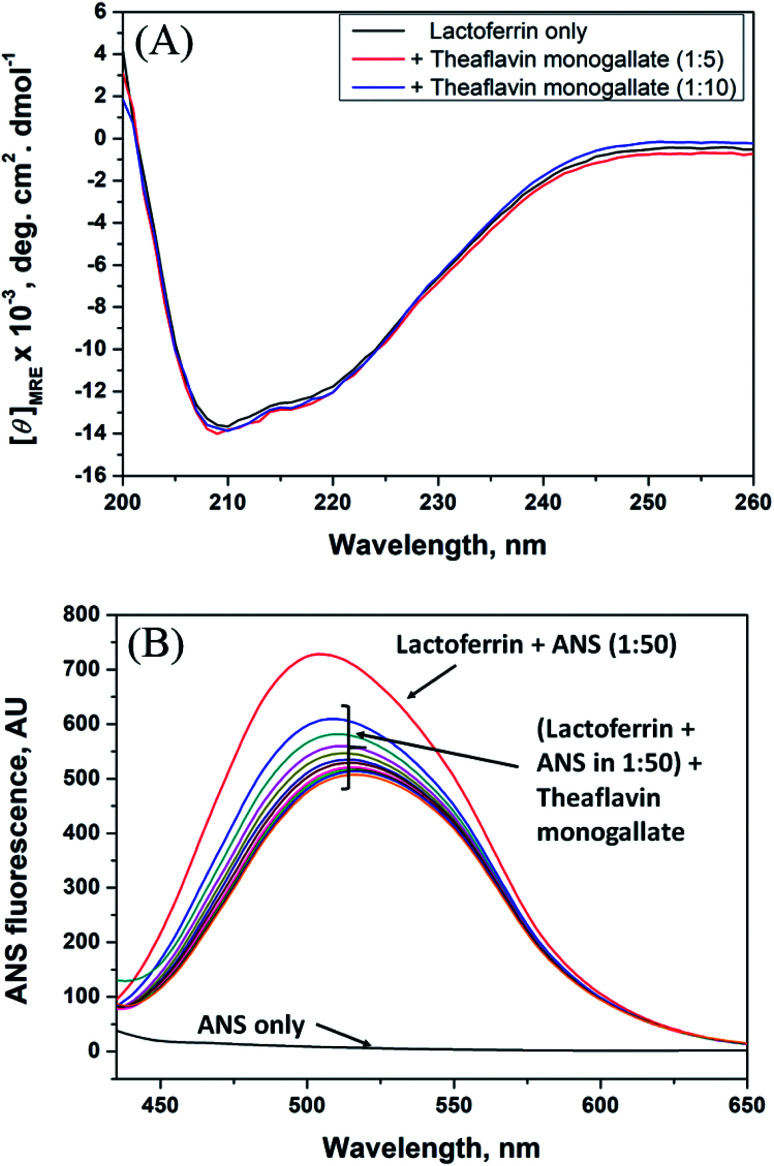
Conformational changes in lactoferrin due to the binding of theaflavin monogallate. (A) Far-UV CD spectra of lactoferrin (5 μM) in the absence and presence of theaflavin monogallate at 1 : 5 and 1 : 10 molar ratios, and (B) ANS fluorescence spectra of lactoferrin (5 μM) in the absence and presence of varying theaflavin monogallate concentration (0–15 μM).

Circular dichroism is one of the most sensitive spectroscopic techniques used to determine the secondary structure of proteins. CD spectra of lactoferrin in the absence and presence of theaflavin monogallate are shown in Fig. 2A. The free lactoferrin exhibited two negative peaks, one at 208 nm and the other at 222 nm. These two negative ellipticities are attributed to the α-helical content of proteins.^50^ The data was first converted into mean residue ellipticity (MRE) using eqn (8).^51^8
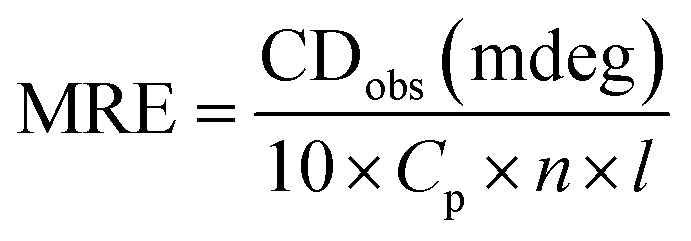
where *C*_p_ is the molar concentration of protein, *n* is the number of amino acids, and *l* is the path length of the cuvette. The percentage of α-helix was calculated from MRE using eqn (9).^51^9
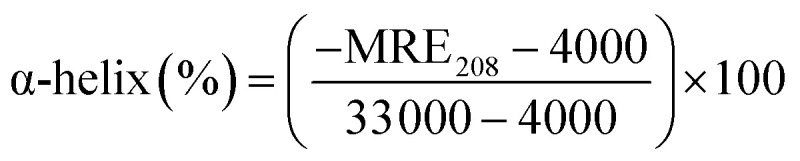
where MRE_208_ is MRE at 208 nm, 4000 is MRE of the random coil conformation, and β-form at 208 nm, and 33 000 is MRE of pure α-helix at 208 nm.

The presence of theaflavin monogallate slightly increased the ellipticity at both negative peaks. The increase in ellipticity indicates a slight increase in the α-helical content of lactoferrin. The amount of α-helix in free lactoferrin was 32.04% which increased to 33.04% and 33.72% after the addition of theaflavin monogallate in 1 : 5 and 1 : 10 molar ratios, respectively. Epigallocatechin gallate (EGCG) changed the conformation of albumin and increase the α-helical content has been earlier reported.^36^ Moreover, Roy *et al.* (2013) also demonstrated that polyphenol (genistein) caused the similar increase in α-helical content of albumin.^52^ Contrary to our results, theaflavins has been shown to decrease the α-helical content of alpha-glucosidase and albumin.^36,53^ The dissimilarity in the results could be due to low concentration of theaflavins, incubation time and different structure of proteins and ligand.

### 3.5. 8-Anilino-1-naphthalene sulfonic acid (ANS) fluorescence assay

ANS fluorescence assay was performed to analyze the effect of theaflavin monogallate on the hydrophobic domains or patches of lactoferrin. ANS is a fluorescent probe that gives negligible fluorescence when present in a polar solution. However, ANS gives strong fluorescence when bound to the hydrophobic domains of protein.^54^ The ANS fluorescence emission spectra of lactoferrin in the absence and presence of theaflavin monogallate are presented in Fig. 2B. Free ANS did not produce much fluorescence in the solution. In contrast, the addition of lactoferrin to ANS solution resulted in remarkable enhancement of the ANS fluorescence showing the attachment of ANS to the hydrophobic domains of lactoferrin. The addition of theaflavin monogallate resulted in a gradual decrease of the ANS fluorescence; saturated at 15 μM theaflavin monogallate. The results indicate that binding of theaflavin monogallate competed for the binding site of ANS. Moreover, this also shows the burial of the hydrophobic domain of lactoferrin on the addition of theaflavin monogallate.

### 3.6. Förster resonance energy transfer (FRET) analysis

**Fig. 3:**
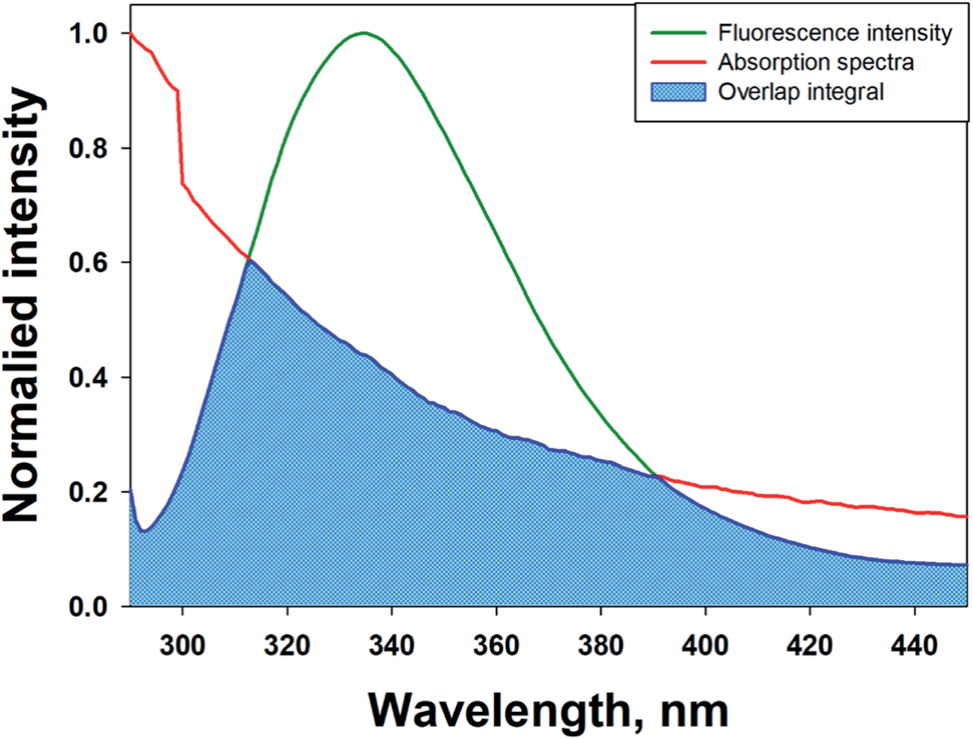
Forster resonance energy transfer (FRET) plot depicting the overlap between the fluorescence intensity of lactoferrin (5 μM) and the absorption spectrum of theaflavin monogallate (10 μM).

FRET is a phenomenon in which the transfer of energy occurs when two molecules interact with each other. This phenomenon is used to determine the energy transfer efficiency and distance between the two interacting molecules. FRET only occurs when the donor molecule's emission spectrum overlaps with the absorption spectrum of the acceptor molecule. For a successful FRET, donor and acceptor molecules must remain in the proximity of less than 10 nm; the donor molecule must have a high quantum yield, and there should be a proper orientation of transition dipole moment of both molecules.^55^ If there is considerable overlap between the acceptor molecule's absorption spectrum and the donor molecule's emission spectrum, then there is a probability of energy transfer between the interacting molecules.^56^ The spectral overlap of fluorescence emission of lactoferrin and absorption spectrum of theaflavin monogallate is shown in Fig. 3. The amount of energy transfer is directly proportional to the degree of spectral overlap. Various parameters of FRET were calculated using eqn (10)–(12).^57,58^10
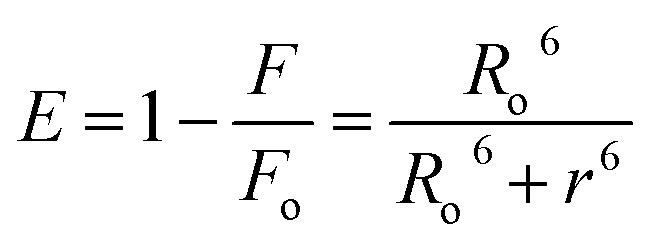

11 *R*_o_^6^ = 8.79 × 10^−25^*K*^2^*n*^−4^*ϕJ*

12
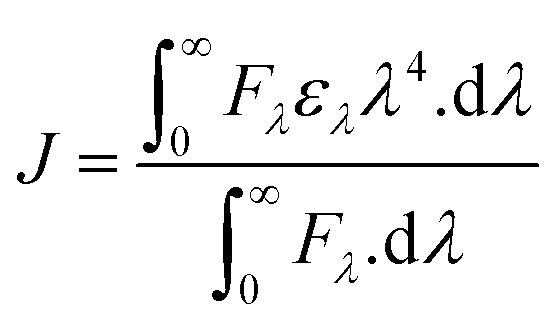
where *F*_o_ and *F* are the fluorescence intensities of lactoferrin in the absence and presence of theaflavin monogallate; *R*_o_ is the critical distance at 50% transfer efficiency, *r* is the distance between donor and acceptor molecules, *K*^2^ is orientation factor (2/3), *ϕ* is fluorescence quantum yield of the donor (0.118), *n* is the refractive index of the medium (1.336), *J* is the degree of spectral overlap, *F*(*λ*) is the fluorescence intensity of donor in wavelength ranging from *λ* to *λ* + Δ*λ*, and *ε*(*λ*) is molar absorptivity of acceptor at *λ*. The parameters for the interaction of theaflavin monogallate to lactoferrin obtained using FRET are enlisted in Table 3.

**Table 3:** FRET parameters for camel milk lactoferrin and theaflavin monogallate interaction

*J* (M^−1^ cm^−3^)	*R*_o_ (nm)	*r* (nm)	*E*_FRET_
1.20 × 10^−14^	2.54	2.44	49.6%

*J* and *R*_o_ values were found to be 1.20 × 10^−14^ cm^3^ M^−1^ and 2.54 nm, respectively. The distance between theaflavin monogallate and lactoferrin was found to be 2.44 nm, and the energy transfer efficiency was obtained found as 49.6%. These values indicate a high probability of energy transfer from lactoferrin to theaflavin monogallate. As per the non-radiative energy transfer theory of Förster, the *R*_o_ and *r* values lies below 10 nm.^59^ Therefore, the values of *R*_o_ and *r* confirms a non-radiative energy transfer mechanism occurring between lactoferrin and theaflavin monogallate. Moreover, FRET parameters also validate that there was static quenching mechanism energy transfer that contributed to the decrease of fluorescence intensity lactoferrin.^60^

### 3.7. Molecular docking

**Fig. 4:**
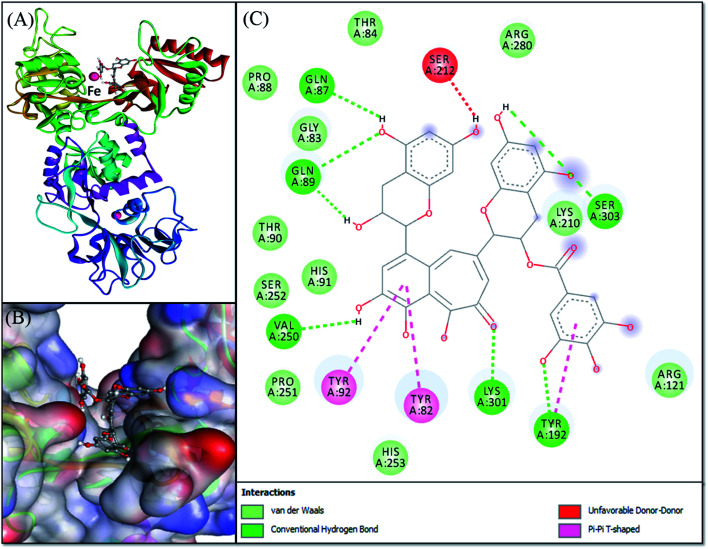
Molecular docking of theaflavin monogallate with lactoferrin. (A) Cartoon representation of theaflavin monogallate (balls and stick model) binding with lactoferrin, (B) binding of theaflavin monogallate at the central cavity of lactoferrin, and (C) 2D depiction of lactoferrin interacting with theaflavin monogallate and the nature of forces involved in stabilizing lactoferrin–theaflavin monogallate complex.

Molecular docking was performed to obtain a closer insight into the binding of theaflavin monogallate to lactoferrin. Such studies provide detailed information regarding the nature of forces involved in interaction and the amino acids interacting with the ligand. The lowest binding energy docked complex of theaflavin monogallate with lactoferrin is shown in Fig. 4. The details of interacting amino acid residues and the nature of forces involved in the interaction are enlisted in Table 4. The binding energy for the interaction of theaflavin monogallate with lactoferrin was −9.7 kcal mol^−1^, which corresponds to the binding affinity of 2.03 × 10^6^ M^−1^. Theaflavin monogallate formed seven hydrogen bonds with Gln87, Gln89, Tyr192, Val250, Lys301, and Ser303 of lactoferrin, while Tyr82, Tyr92, and Tyr192 interacted with theaflavin monogallate *via* three hydrophobic interactions (Pi–Pi T-shaped). Also, several residues such as Gly83, Thr84, Pro88, Thr90, His91, Arg121, Lys210, Pro251, Ser252, His253, and Arg280 formed van der Waals' interactions. Moreover, Ser212 was involved in an unfavorable interaction with theaflavin monogallate.

**Table 4:** Molecular docking parameters for the interaction between camel milk lactoferrin and theaflavin monogallate

Donor atom–acceptor atom pair	Distance (Å)	Nature of interaction	Docking energy, Δ*G* (kcal mol^−1^)	Binding affinity, *K*_d_ (M^−1^)
GLN89:HN–LIG:O	2.92864	Hydrogen bond	−9.7	2.03 × 10^6^
TYR192:HH–LIG:O	2.69661	Hydrogen bond		
LYS301:HZ1–LIG:O	2.46215	Hydrogen bond		
LIG:H–SER303:O	2.50962	Hydrogen bond		
LIG:H–GLN89:O	1.73359	Hydrogen bond		
LIG:H–GLN87:O	2.33993	Hydrogen bond		
LIG:H–VAL250:O	2.41777	Hydrogen bond		
TYR82–LIG	4.65393	Hydrophobic (Pi–Pi T-shaped)		
TYR92–LIG	4.74263	Hydrophobic (Pi–Pi T-shaped)		
TYR192–LIG	5.12632	Hydrophobic (Pi–Pi T-shaped)		

### 3.8. Molecular dynamics (MD) simulation

**Fig. 5:**
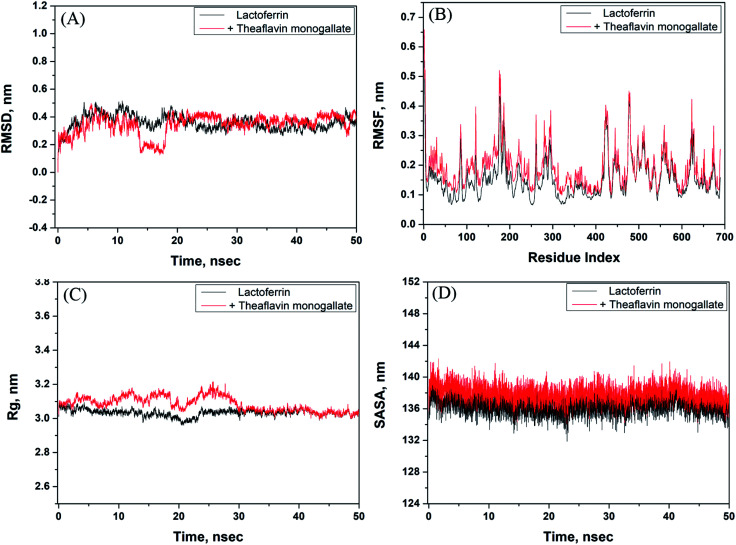
Molecular dynamics (MD) simulation of lactoferrin and theaflavin monogallate interaction. (A) Variation in RMSD (root mean square deviation) of lactoferrin alone and lactoferrin–theaflavin monogallate complex as a function of simulation, (B) RMSF (root mean square fluctuation) in lactoferrin in the absence and presence of theaflavin monogallate, (C) dependency of *R*_g_ (radius of gyration) of lactoferrin alone and lactoferrin–theaflavin monogallate complex as a function of simulation, and (D) variation in SASA (solvent accessible surface area) of lactoferrin alone and lactoferrin–theaflavin monogallate complex as a function of simulation.

The stability of the theaflavin monogallate–lactoferrin complex was further studied by molecular dynamics (MD) simulation. The protein–ligand complex is dynamic in nature; therefore, its stability was assessed by simulating at the physiological conditions (Fig. 5). The complex obtained in molecular docking was used as initial conformation, and MD simulation was performed for 50 ns. The RMSD (root mean square variation) of lactoferrin and its complex with theaflavin monogallate with respect to the original frame is presented in Fig. 5A. There was a little deviation in RMSD of the complex from 0 to 20 ns, which at a later part (20–50 ns) of the simulation gets stabilized. The small deviation might be due to the entry of theaflavin monogallate into the cavity of lactoferrin. The mean RMSD values of lactoferrin and lactoferrin–theaflavin monogallate systems for 20–50 ns were 0.3693 and 0.3781 nm, respectively. These results confirmed the formation of a stable complex between lactoferrin and theaflavin monogallate. Moreover, RMSF (root mean square fluctuation) along the lactoferrin chain were calculated to analyze the local conformational alterations caused by the binding of theaflavin monogallate (Fig. 5B). It was found that the fluctuation in residues of lactoferrin–theaflavin monogallate complex exhibited a similar fluctuation pattern as exhibited by lactoferrin alone. The results confirm that no significant structural changes occurred in lactoferrin due to the binding of theaflavin monogallate.

The radius of gyration (*R*_g_) of lactoferrin and its complex was also calculated to ascertain the structural stability as a function of simulation time (Fig. 5C). The *R*_g_ of lactoferrin and lactoferrin–theaflavin monogallate complex remained constant approximately at 3.01 and 3.02 nm, indicating that lactoferrin did not undergo any significant conformational alterations. Finally, the stability of the complex between theaflavin monogallate and lactoferrin was further validated by computing the solvent-accessible molecular surface area (SASA) as a function of simulation time (Fig. 5D). It was found that SASA of lactoferrin and complex remained constant within limits. The average values of SASA of lactoferrin alone and in complex with theaflavin monogallate were estimated as 135.45 and 136.58 nm^2^, respectively. Thus, the results of molecular dynamics simulations indicate that theaflavin monogallate formed a stable complex with lactoferrin.

## 4. Conclusion

The findings of this study provide detailed insights into the interaction between theaflavin monogallate and lactoferrin. Theaflavin monogallate interacted with lactoferrin with a moderate binding affinity. The mode of fluorescence quenching was found to be static. The values of various thermodynamic parameters showed that a change in entropy drove the binding. The interaction between lactoferrin and theaflavin monogallate resulted in forming a stable complex in which α-helical content was not altered. The conformation of lactoferrin remained nearly uncaged, and energy transfer from lactoferrin to theaflavin monogallate was efficient. Theaflavin monogallate binds the central cavity of lactoferrin and interacts with key amino acid residues. Hydrogen bonding and hydrophobic interactions were predominant forces in the formation of a stable protein–polyphenol complex. The parameters of molecular dynamics simulations further validated the stability of theaflavin monogallate–lactoferrin complex. These findings are significant in understanding the nature and mechanism of interaction for the binding of theaflavin monogallate with lactoferrin at the molecular level.

## Conflicts of interest

There are no conflicts to declare.

## Supplementary Material

RA-011-D1RA03256A-s001
