# Supplementary material for: Elucidation of molecular interactions of theaflavin monogallate with camel milk lactoferrin: detailed spectroscopic and dynamic simulation studies
Source: RSC Adv. 2021 Aug 4;11(43):26710–20. doi: 10.1039/d1ra03256a (PMC9037349; doi:10.1039/d1ra03256a)
Supplement: RA-011-D1RA03256A-s001 [file RA-011-D1RA03256A-s001.pdf]

Electronic Supplementary Material (ESI) for RSC Advances.
This journal is © The Royal Society of Chemistry 2021

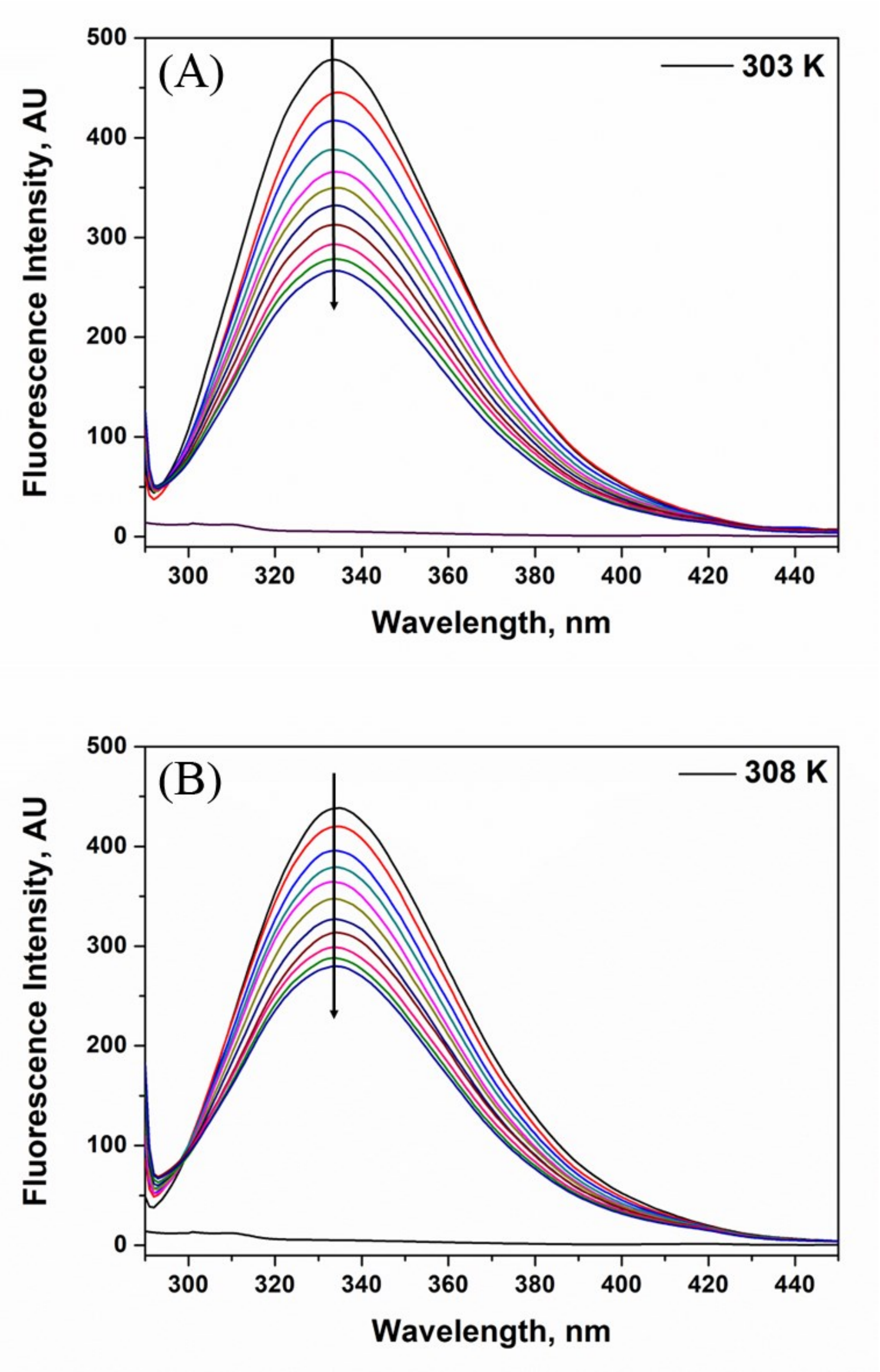

Supplementary Fig 1: Quenching in the fluorescence intensity of lactoferrin (5 μM) due to the binding of theaflavin monogallate (0-15 μM) at (A) 303 K, and (B) 308 K.
